# Football, fossil fuels, and public health in the era of climate crisis

**DOI:** 10.1097/EE9.0000000000000502

**Published:** 2026-07-21

**Authors:** Manolis Kogevinas, Neil Pearce

**Affiliations:** a Environment and Health over the Lifecourse Program, ISGlobal, Barcelona, Spain; b Department of Medical Statistics, London School of Hygiene and Tropical Medicine, London, United Kingdom

**Keywords:** Climate change, Commercial determinants of health, Fossil fuels, Sports sponsorship, Environmental health

## Abstract

Global professional football (soccer in the United States) is increasingly integrated with fossil fuel–derived capital through club ownership, expansive sponsorship deals, and tournament hosting. While often discussed in ethical or political terms, these developments have received limited attention from an environmental health perspective. This commentary examines the growing alignment between fossil fuel-derived wealth and global football, focusing primarily on club ownership, while situating it within a broader ecosystem of commercial and geopolitical influence. We argue that these relationships function as a commercial determinant of health by shaping social norms, legitimizing carbon-intensive industries, and delaying policy action on climate change. Drawing parallels to the historical regulation of tobacco sponsorship, we highlight how such dynamics contribute to the persistence of exposures that drive climate-related health risks. Climate change is the great public health challenge of the 21st century, just as tobacco was in the 20th century. Greater scrutiny, transparency, and leadership from the public health community with regard to fossil fuel–derived funding of sport are warranted. It is no longer acceptable for major sporting bodies and events to accept tobacco industry funding. It should also be unacceptable for them to accept fossil fuel–derived funding.

**What this study adds**

This commentary positions fossil fuel sponsorship and ownership in professional football as an underrecognized commercial determinant of health and environmental exposure. It contributes to the environmental epidemiology literature by linking sport-related fossil fuel promotion with pathways that normalize polluting industries, weaken climate action, and indirectly contribute to climate-related health burdens. By drawing parallels with tobacco sponsorship, the paper highlights the potential public health relevance of regulating such partnerships. We submit this commentary to Environmental Epidemiology because it addresses the intersection of climate change, corporate influence, and population health, an area of growing importance for environmental health research and policy.

“Sport can create hope where once there was only despair”—Nelson Mandela

We can only agree with Nelson Mandela. Sport can be inspiring, uplifting, and can create hope. But we have to confess something. When Manchester City plays against Real Madrid, we are not sure which team we want to win. Under normal circumstances, the answer should be simple. We admire Manchester City’s football. We admire even more the mind behind it, Pep Guardiola; his teams have redefined the game, intelligent, fluid, almost artistic.

And yet, we hesitate. Because for everything else we believe in, about climate change, environmental responsibility, and the role of power, we cannot ignore what Manchester City represents: a project backed by the United Arab Emirates. We find ourselves in the uncomfortable position of admiring the football while questioning the economic and political structures that make it possible. This is not just a personal dilemma. It reflects a broader tension within modern football and global environmental health and positions fossil fuel sponsorship and ownership in professional football as an underrecognized commercial determinant of environmental exposures and health.

## Football and fossil fuel capital

While fans are encouraged to judge the game solely on its sporting merits, the “neutrality” of sport is increasingly illusory. Football is one of the most visible global cultural industries, reaching billions of football fans, and has become increasingly intertwined with capital originating from fossil fuel–dependent economies. The fossil fuel industry includes companies involved in the extraction, refining, and sale of oil, gas, and coal. The fossil fuel industry is estimated to currently invest at least $5.6 billion annually in global sports sponsorship across more than 250 active deals,^1,2^ with football accounting for the largest share. This integration occurs through three primary mechanisms: the ownership of elite clubs, sponsorship and advertising agreements, and the hosting of major international tournaments. From an environmental health perspective, these relationships are not neutral: they contribute to the social normalization and political entrenchment of industries that drive climate change, widely recognized as a major determinant of population health. Although these influences are indirect, they shape social norms and regulatory environments that affect population-level exposures to climate-sensitive hazards, including heat, air pollution, and extreme weather events, all of which are directly relevant to environmental epidemiology.

## Ownership, sponsorship, and global influence

Several prominent European football clubs are currently owned by state-linked entities from major fossil fuel–producing countries. Manchester City is controlled by Abu Dhabi interests, Paris Saint-Germain by Qatar Sports Investments, and Newcastle United by Saudi Arabia’s Public Investment Fund. These ownership structures are not merely financial arrangements; they are embedded within broader strategies of international positioning, where sport serves as a highly visible platform for projecting influence and legitimacy.

Sponsorship and advertising provide an additional and pervasive channel of engagement. Major football clubs and competitions maintain partnerships with airlines, energy companies, and other corporations linked to fossil fuel economies. Stadium naming rights, shirt sponsorships, and broadcast partnerships ensure continuous visibility of these actors.

The hosting of major tournaments reinforces these dynamics at a global scale. Recent and upcoming FIFA World Cups have been awarded to Qatar (2022) and Saudi Arabia (2034), both major fossil fuel producers. Such events provide opportunities not only for economic activity but are particularly effective vehicles for shaping international perception.

The entanglement of sport and state power became particularly visible during the 2022 FIFA World Cup final when Lionel Messi was draped in a traditional Qatari garment/bisht by the Emir before lifting the trophy (http://bit.ly/3RsLBA8). This act merged the most iconic image in global sport, Messi at the peak of his career, with the identity of the host state, making it difficult to sustain the idea that sport and power can be cleanly separated.^3,4^

The case of Manchester City under the management of Pep Guardiola (and recently of Paris Saint-Germain under Luis Enrique) further illustrates the normalization of these dynamics within elite football. Both teams’ style of play has been widely praised for their technical and tactical sophistication, contributing to their global appeal. Yet their success is inseparable from a system financed by fossil fuel–derived capital. This juxtaposition reflects a broader systemic condition in which sporting excellence and environmentally consequential funding sources are closely intertwined.

## Sportswashing as a commercial determinant of health

These developments are often described as “sportswashing,” defined as the use of sport to project soft power, enhance international legitimacy, and influence public narratives. Fossil fuel industries and the states whose economies depend on them play a central role in driving climate change.^5^ Of course, the fossil fuel industry is not the only potentially hazardous industry promoted through the funding of football. For example, gambling sponsorship is another potentially hazardous industry promoted through football, albeit with notable examples in several European leagues. However, we consider that fossil fuel sponsorship raises a higher level of ethical concern, as well as dwarfing the financial footprint of other sponsorships from other hazardous industries. For example, stadium naming rights by major airlines linked to fossil fuels demonstrate pervasive indirect promotion. The continued prominence of these actors within sport raises similar concerns regarding normalization and public perception as those raised for the tobacco industry.^6^ At a global scale, fossil fuel sportswashing acts as a powerful commercial determinant of health by influencing exposures, behaviors, and policy environments. These dynamics operate through indirect but well-established pathways. Sponsorship enhances legitimacy and shapes public narratives, weakening regulatory momentum and delaying policy action. This sustains emissions and amplifies downstream health risks, including heat-related morbidity and mortality, air pollution–related disease, and climate-sensitive infections.^5^

## Is fossil fuel capital structurally necessary?

A commonly invoked justification for such financial arrangements is that elite clubs require continuous investment to remain competitive. However, football operates within a self-reinforcing economic system where increased revenues are typically translated into higher player salaries, transfer fees, and associated costs. Financial expansion does not resolve structural instability; it merely reproduces it at a higher level of expenditure.

## Football in a warming world

Ironically, football is increasingly vulnerable to the very climate consequences these industries drive. Rising temperatures pose heat-related risks to players and spectators, while extreme weather disrupts competition calendars and infrastructure.^7^ By serving as a beneficiary of fossil wealth while suffering its effects, football exemplifies the contradictions of sports in a warming world, posing risks of heat illness for players and spectators, disrupting physical activity patterns, and undermining the broader health benefits associated with sport participation.

## The public health parallel: tobacco and fossil fuels

Scientific societies have previously played an important role in establishing ethical standards in relation to industry influence, most notably in the case of tobacco. As evidence on health harms accumulated, such sponsorship became increasingly restricted. The tobacco industry famously used sports sponsorship to associate its products with vitality and success, exemplified by the Winfield Cup in rugby or the pervasive presence of Marlboro in Formula 1. As the health consequences became undeniable, these practices were restricted by international frameworks like the World Health Organization Framework Convention on Tobacco Control.^8^ The transition from total saturation to a total ban offers numerous critical policy precedents for fossil fuel regulation in sports. The 1988 Calgary Winter Olympics, for example, marked a major moment for sport-led public health policy. Following pressure from athletes, most notably Canadian skier Steve Podborski, who refused to ski through gates bearing tobacco logos, the Canadian Olympic Committee banned tobacco marketing. As with tobacco, sports sponsorship does not directly cause disease but shapes the social and regulatory environments that determine exposure at a population level.

The recent collective decision by Premier League clubs to phase out front-of-shirt gambling sponsorship by 2026 provides a clear regulatory roadmap.^9^ While clubs argue that such “controversial” capital is essential for survival, the betting ban demonstrates that when social harm is recognized, the industry can, and does, pivot. However, a glaring inconsistency remains: while gambling is being restricted due to its impact on individual health, fossil fuel sponsorship, which drives the global climate crisis and threatens population-level health, remains largely unregulated.

## The role of environmental health professionals

From the perspective of environmental and public health professionals, these developments raise questions that extend beyond sport. Potential actions include advocating for greater transparency in ownership and sponsorship structures, developing guidelines addressing conflicts of interest, and engaging with sporting organizations to align their commercial practices with global sustainability goals.

Engaging with these issues does not require rejecting sport, but rather recognizing sport’s role within systems that shape environmental exposures. Football, like other global cultural platforms, reflects the economic and political structures within which it operates. The discomfort we feel when watching a game financed by the drivers of the climate crisis is not a personal quirk; it is a signal of a systemic misalignment. Recognizing these tensions is about understanding football’s place within the systems that ultimately determine population health. For environmental epidemiology, this implies extending the scope of inquiry beyond proximal exposures to include the upstream commercial and political forces that shape them.

Climate change is the great public health challenge of the 21st century, just as tobacco was in the 20th century.^10^ It is no longer acceptable for major sporting bodies and events to accept tobacco industry funding. It should also be unacceptable for them to accept fossil fuel-derived funding.

## ABOUT THE AUTHORS

Manolis Kogevinas, MD, PhD, is Professor Emeritus of Epidemiology at Barcelona Institute for Global Health (ISGlobal). He has held positions at the International Agency for Research on Cancer (IARC) and the University of Crete. His research focuses on environmental and occupational epidemiology, climate and health, and the health impacts of complex environmental exposures. He is a past President of the International Society for Environmental Epidemiology and has contributed to international evaluations of environmental and occupational carcinogenic hazards. Neil Pearce, PhD, is Professor of Epidemiology and Biostatistics at the London School of Hygiene & Tropical Medicine. His research has advanced epidemiologic methods and their application to environmental and occupational health, asthma, and chronic disease epidemiology. He is internationally recognized for his contributions to causal inference, study design, and the interpretation of epidemiologic evidence, and has served in leadership roles within the International Epidemiologic Association and other international scientific organizations.
